# Carbonyl sulfide (COS) as a tracer for canopy photosynthesis, transpiration and stomatal conductance: potential and limitations

**DOI:** 10.1111/j.1365-3040.2011.02451.x

**Published:** 2012-04

**Authors:** Georg Wohlfahrt, Federico Brilli, Lukas Hörtnagl, Xiaobin Xu, Heinz Bingemer, Armin Hansel, Francesco Loreto

**Affiliations:** 1 Institute of Ecology, University of InnsbruckSternwartestr. 15, 6020 Innsbruck, Austria; 2 Ionicon Analytik GmbHEduard-Bodem-Gasse 3, 6020 Innsbruck, Austria; 3 Key Laboratory for Atmospheric Chemistry, Centre for Atmosphere Watch & Services, Chinese Academy of Meteorological Sciences, China Meteorological AdministrationZhongguancun Nandajie 46, Beijing 100081, China; 4 Institute for Atmospheric and Environmental Sciences, J.W. Goethe UniversityAltenhöferallee 1, 60438 Frankfurt am Main, Germany; 5 Institute of Ion Physics and Applied Physics, University of InnsbruckTechnikerstr. 25, 6020 Innsbruck, Austria; 6 Institute for the Protection of Plants, National Research CouncilVia Madonna del Piano 10, 50019 Sesto Fiorentino, Firenze, Italy

**Keywords:** carbon dioxide, gross photosynthesis, internal conductance, net photosynthesis, water vapour

## Abstract

The theoretical basis for the link between the leaf exchange of carbonyl sulfide (COS), carbon dioxide (CO_2_) and water vapour (H_2_O) and the assumptions that need to be made in order to use COS as a tracer for canopy net photosynthesis, transpiration and stomatal conductance, are reviewed. The ratios of COS to CO_2_ and H_2_O deposition velocities used to this end are shown to vary with the ratio of the internal to ambient CO_2_ and H_2_O mole fractions and the relative limitations by boundary layer, stomatal and internal conductance for COS. It is suggested that these deposition velocity ratios exhibit considerable variability, a finding that challenges current parameterizations, which treat these as vegetation-specific constants. COS is shown to represent a better tracer for CO_2_ than H_2_O. Using COS as a tracer for stomatal conductance is hampered by our present poor understanding of the leaf internal conductance to COS. Estimating canopy level CO_2_ and H_2_O fluxes requires disentangling leaf COS exchange from other ecosystem sources/sinks of COS. We conclude that future priorities for COS research should be to improve the quantitative understanding of the variability in the ratios of COS to CO_2_ and H_2_O deposition velocities and the controlling factors, and to develop operational methods for disentangling ecosystem COS exchange into contributions by leaves and other sources/sinks. To this end, integrated studies, which concurrently quantify the ecosystem-scale CO_2_, H_2_O and COS exchange and the corresponding component fluxes, are urgently needed.

We investigate the potential of carbonyl sulfide (COS) for being used as a tracer for canopy net photosynthesis, transpiration and stomatal conductance by examining the theoretical basis of the link between leaf COS, carbon dioxide (CO2) and water vapour (H2O) exchange. Our analysis identifies several limitations that need to be overcome to this end, however at present we lack appropriate ecosystem-scale field measurements for assessing their practical significance. It however appears that COS represents a better tracer for CO2 than H2O. Concurrent measurements of ecosystem scale COS, CO2 and H2O exchange are advocated.

## INTRODUCTION

Canopy net photosynthesis, transpiration and stomatal conductance are key conceptual terms in most contemporary models of ecosystem carbon and water cycling (Sitch *et al*. 2008). While net photosynthesis, transpiration and stomatal conductance can be quantified accurately with enclosures at the leaf scale [e.g. von Caemmerer & Farquhar (1981), but see Rodeghiero, Niinemets & Cescatti (2007)], obtaining reliable estimates at the canopy scale is much more difficult. Scaling up net photosynthesis, transpiration and stomatal conductance measured in leaf enclosures to the canopy scale requires minimum knowledge on (1) the response of leaf gas exchange rates to environmental drivers; (2) how this response changes with depth in the canopy; (3) the vertical variation of environmental drivers within the plant canopy; and (4) the vertical distribution of the assimilating/transpiring plant area (Kruijt, Ongeri & Jarvis 1997; Wohlfahrt *et al*. 2010). Transpiration of individual trees can be directly quantified by sap flux methods; however, up-scaling methods are again needed to turn these measurements into canopy transpiration (Wilson *et al*. 2001). Enclosures that include entire ecosystem are prone to artefacts due to modifications of the environmental conditions (Dore *et al*. 2003). In addition, ecosystem enclosures yield the net ecosystem CO_2_ and H_2_O exchange, that is, they are unable to partition between canopy net photosynthesis/transpiration and CO_2_/H_2_O fluxes from/to the soil and other ecosystem components. This drawback also holds for micrometeorological techniques such as the eddy covariance method, which, however has the advantage of being unobtrusive and able to provide near-continuous long-term flux data (Baldocchi, Hicks & Meyers 1988; Baldocchi 2003). In order to recover canopy net photosynthesis and transpiration from net ecosystem fluxes of CO_2_ and H_2_O, it is necessary to concurrently quantify/estimate the confounding CO_2_ and H_2_O fluxes – a non-trivial task in particular for CO_2_, which has multiple sources in an ecosystem. Converting estimates of canopy transpiration to stomatal conductance in turn is fraught with problems due to difficulties with the correct specification of the vapour gradient between the transpiring surface and ambient air (Magnani *et al*. 1998). As a consequence, available estimates of canopy net photosynthesis, transpiration and stomatal conductance are inherently uncertain, which in turn translates into uncertain model parameterizations and predictions.

Recently, several authors have advocated measurements of COS exchange to provide independent constraints on canopy net photosynthesis in particular (Sandoval-Soto *et al*. 2005; Montzka *et al*. 2007; Campbell *et al*. 2008; Brugnoli & Calfapietra 2010; Seibt *et al*. 2010; Stimler *et al*. 2010a, 2011), as well as on canopy transpiration and stomatal conductance (Seibt *et al*. 2010). The rationale for these proposals derives from both leaf and (very few) ecosystem flux measurements, which show a high degree of co-variation between the net exchange rates of CO_2_, H_2_O and COS (Xu, Bingemer & Schmidt 2002; Sandoval-Soto *et al*. 2005; Stimler *et al*. 2010a).

Given the promising possibility of quantifying ecosystem-scale COS exchange by using the eddy covariance method in combination with new analytical techniques (Graus, Müller & Hansel 2010; Stimler, Nelson & Yakir 2010b), in order to better constrain canopy photosynthesis, transpiration and stomatal conductance, the objective of the present paper is: (1) to review the mechanistic link between leaf- and ecosystem-scale CO_2_, H_2_O and COS fluxes; (2) to critically evaluate the assumptions required for estimating canopy net photosynthesis, transpiration and stomatal conductance from COS exchange measurements; and finally (3) to indicate areas of future research.

## THE LINK BETWEEN LEAF CO_2_, H_2_O AND COS EXCHANGE

**Fig. 1:**
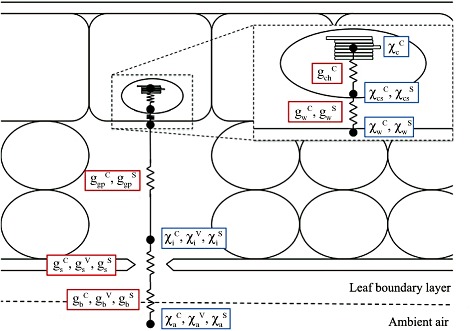
Schematic illustration of the diffusion pathways of CO_2_, H_2_O and COS into/out of a leaf. Blue panels represent mole fractions; red panels are conductances using abbreviations introduced in the text. Additional abbreviations include: *g*_gp_^C^ and *g*_gp_^S^ referring to gas phase conductances for CO_2_ and COS in the intercellular space; *g*_w_^C^ and *g*_w_^S^ (including cell wall, plasma membrane and cytosol) and χ_w_^C^ and χ_w_^S^ referring to cell wall conductances and mole fractions of CO_2_ and COS, respectively; χ_cs_^C^ and χ_cs_^S^ referring to chloroplast surface mole fractions of CO_2_ and COS; *g*_ch_^C^ referring to the chloroplast conductance to CO_2_ and χ_c_^C^ referring to the chloroplast CO_2_ mole fraction. Following Stimler *et al*. (2010a), who concluded CA to be effectively located at the chloroplast surface, the end point for diffusion of COS is placed at the inside of the chloroplast surface.

We begin our assessment with a review of the equations describing the diffusive flux of CO_2_, H_2_O and COS in/out of leaves, as sketched in Fig. 1. Leaf net photosynthesis (*F*_l_^C^) is given as


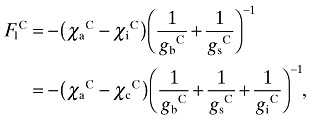
(1)

leaf transpiration (*F*_l_^V^) as


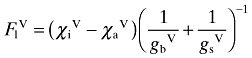
(2)

and the leaf net flux of COS (*F*_l_^S^) as


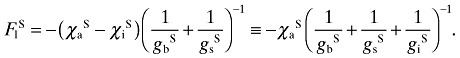
(3)

Here, χ refers to mole fractions (subscripts: a … ambient air, i … intercellular space, c … chloroplast) and *g* to conductances (subscripts: b … boundary layer, s … stomata, i … internal). Note that we employ a sign convention by which fluxes directed into the leaf have a negative sign. The boundary layer and stomatal conductances may be inter-converted between COS, CO_2_ and H_2_O based on their diffusivity (*D*) ratios, that is,


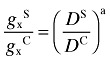
(4)

where the exponent ‘a’ takes a value of 1 for *g*_s_ (i.e. molecular diffusion) and 0.67 for *g*_b_ (i.e. forced convection which typically applies in well-ventilated leaf enclosures) (Campbell & Norman 1998). *g*_s_^S^ and *g*_b_^S^ may thus be converted to their CO_2_ (H_2_O) counterparts by multiplication with *ca.* 1.21 (2.00) and 1.14 (1.59), respectively (Seibt *et al*. 2010; Stimler *et al*. 2010a).

While Eqns 1 and 2 are well established (von Caemmerer & Farquhar 1981), Eqn 3 merits further explanation: the endpoint of the diffusion gradient for COS is the location of the enzyme CA, which appears to be available throughout the plasma membrane, cytosol, chloroplast envelope and stroma (Evans *et al*. 2009). Stimler *et al*. (2010a), following Gillon & Yakir (2000), concluded CA to be effectively located at the chloroplast surface, an assumption we graphically followed in Fig. 1. CA, which has an extremely high sensitivity to COS [larger by a factor of 1000 compared with CO_2_; Protoschill-Krebs, Wilhelm & Kesselmeier (1996)], is responsible for the hydration of COS, as nicely demonstrated by Stimler *et al*. (2011) with CA-deficient antisense lines of C_3_ and C_4_ plants. The hydration of COS is essentially a one-way reaction whereby one molecule H_2_S and CO_2_ are generated for each hydrated molecule COS (Protoschill-Krebs *et al*. 1996). As emissions of COS out of leaves have not been reported even at very low ambient COS concentrations (Seibt *et al*. 2010), it is reasonable to assume that the COS mole fraction at the CA reaction site (χ_i_^S^) is much smaller than its ambient concentration. We therefore follow others (Seibt *et al*. 2010; Stimler *et al*. 2010a) in assuming that χ_i_^S^ can be neglected, as has been done on the right-hand side of Eqn 3. As depicted in Fig. 1, the similarity in diffusion pathways of CO_2_ and COS depends on where actually most of COS becomes hydrated by CA, which is somewhere between the plasma membrane and the chloroplast stroma. Additional differences in *g*_i_^S^ and *g*_i_^C^ arise from differing biochemical reaction rates of CA and Rubisco, respectively, which are implicit in their numerical values.

In summary, the diffusion pathway of COS is not identical to, but more similar for CO_2_ than H_2_O (Fig. 1). In the following, we will discuss the implications of these differences for using COS as a tracer for canopy CO_2_ and H_2_O exchange. As previous studies were almost exclusively interested in linking photosynthesis to COS exchange, we first develop the procedure for estimating CO_2_ from COS fluxes and then turn to the subject of water vapour fluxes. We would like to note that the approach outlined below, in contrast to what several studies have suggested (Stimler *et al*. 2010a), allows the quantification of canopy net, but not gross photosynthesis. Gross photosynthesis equals net photosynthesis minus any autotrophic respiration that continues in the light (Larcher 2001), a dissimilatory process with no apparent link to leaf COS uptake.

## ESTIMATING CANOPY NET PHOTOSYNTHESIS FROM COS EXCHANGE MEASUREMENTS

### Linking leaf-level COS and CO_2_ exchange

In order to provide independent estimates of net photosynthesis, leaf COS exchange measurements have to allow eliminating the unknowns in Eqn 1. Given that Eqn 3 contains three unknowns (*g*_b_^S^, *g*_s_^S^ and *g*_i_^S^ assuming *F*_l_^S^ and χ_a_^S^ to be known) and inserting Eqn 3 into Eqn 1, making use of Eqn 4, would add two new unknowns for the one removed, this is however not possible. This problem has been overcome by parameterization of the relationship between COS and CO_2_ fluxes through the ratio of their deposition velocities, that is, the flux, normalized with the ambient concentration, of COS relative to CO_2_ (for negative values of *F*_l_^C^), that is


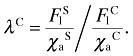
(5)

Based on an estimate of λ^C^ and measurements of χ_a_^C^, χ_a_^S^ and *F*_l_^S^, *F*_l_^C^ (negative values only) may then be simply calculated as (Campbell *et al*. 2008):


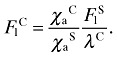
(6)

Up to now, parameterizations of λ^C^ have treated it as a vegetation-type specific constant (Sandoval-Soto *et al*. 2005; Campbell *et al*. 2008; Seibt *et al*. 2010), but how constant is λ^C^ expected to be? In order to answer this question, we rearrange Eqns 1 and 3 to yield the respective deposition velocities, that is


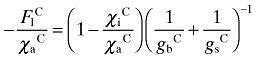
(7)

and


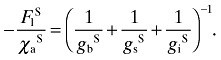
(8)

Making use of the diffusivity ratios (Eqn 4), we now introduce a non-dimensional coefficient β^C^ as


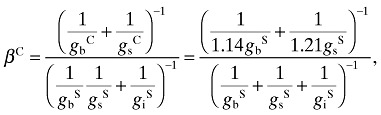
(9)

which when combined with Eqn 5 yields the following expression:


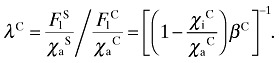
(10)

A similar, albeit less general, expression was derived by Seibt *et al*. (2010). It functionally relates the deposition velocities for CO_2_ and COS and shows that the CO_2_ deposition velocity will, for any given COS deposition velocity, vary with four unknowns: the ratio of intercellular to ambient CO_2_ concentration [cf. fig. 3 in Seibt *et al*. (2010)], *g*_b_^S^, *g*_s_^S^ and *g*_i_^S^, which have been incorporated into our coefficient β^C^ for convenience.

**Fig. 3:**
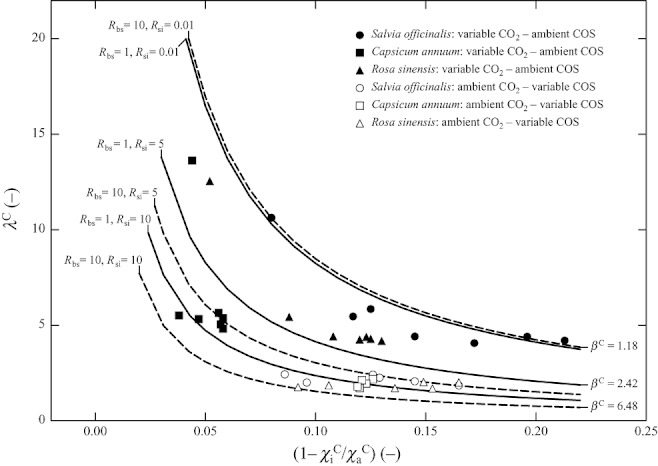
The ratio of COS to CO_2_ deposition velocities (λ^C^) as a function of the intercellular to ambient CO_2_ concentration (1 − χ_i_^C^/χ_a_^C^). Lines represent simulations based on Eqns 9 and 10 for various combinations of boundary to stomatal (*R*_bs_) and stomatal to internal (*R*_si_) COS conductance ratios (the resulting β^C^ values are given in the right lower corner). Symbols represent data digitized from fig. 6 of Stimler *et al*. (2010a). Ambient CO_2_ and COS concentrations refer to 380 µmol mol^−1^ and 500 pmol mol^−1^, respectively. Calculations assumed a constant boundary layer conductance to CO_2_ (1.17 mol m^−2^ s^−1^; fig. 3 in Stimler *et al*. 2010a).

By expressing these three conductances in a mutual fashion as ratios, that is, 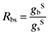 and 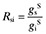, and after some manipulation of Eqn 9, the roles of *g*_b_^S^, *g*_s_^S^ and *g*_i_^S^ in determining β^C^ can be explored, that is


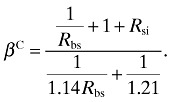
(11)

**Fig. 2:**
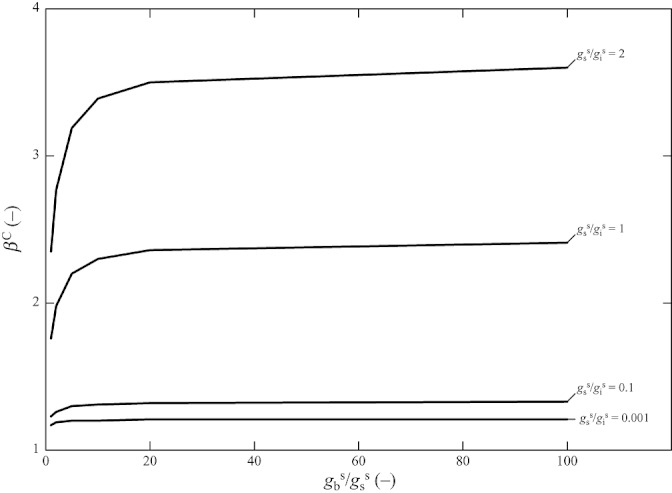
Coefficient β^C^ (Eqn 11) as a function of the relative magnitudes of boundary to stomatal (*g*_b_^s^/*g*_s_^s^) and stomatal to internal (*g*_s_^s^/*g*_i_^s^) conductance for COS.

Two limits of Eqn 11 are useful to explore: the first one relates to the case when *R*_bs_ approaches infinity, as might be observed in a well-ventilated leaf enclosure where *g*_b_ >> *g*_s_. In this case, it can be shown that β^c^ ≡ 1.21 (1 + *R*_si_), that is, β^C^ increases in a linear fashion with the ratio of stomatal to mesophyll conductance with the slope and *y*-intercept equal to 1.21, that is, the *g*_s_^S^/*g*_s_^C^ ratio. The second limit relates to the case of an infinite *g*_i_^s^, that is, *R*_si_ approaches zero. In this case, Eqn 11 reduces to 
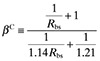
which corresponds to an asymptotic increase from 1.14, that is, the *g*_b_^S^/*g*_b_^C^ ratio, when *R*_bs_ is very small to the previously found limit of 1.21 when *R*_bs_ becomes very large. Assuming physically/physiologically plausible values of 1–100 and 0.001–2 for *R*_bs_ and *R*_si_, respectively, yields β^C^ values in the range of 1.2–3.6 (Fig. 2). Combining these values with χ_i_^C^/χ_a_^C^ ratios typical for C_3_ plants of 0.5–0.8 (Larcher 2001) yields a range of 0.6–4.3 for λ^C^, broadly in correspondence with the spread of 0.4–10.3 reported by Sandoval-Soto *et al*. (2005) in a recent literature survey. Lower χ_i_^C^/χ_a_^C^ ratios of C_4_ plants result in correspondingly lower λ^C^ values (Stimler *et al*. 2011). An example of the actual variability of λ^C^ as a function of the intercellular to ambient CO_2_ concentration and β^C^ is shown in Fig. 3 using data digitized from fig. 6 of Stimler *et al*. (2010a). While part of the variability in λ^C^ relates to changes in the intercellular to ambient CO_2_ concentration ratio (leading to the asymptotic decrease as χ_i_^C^/χ_a_^C^ decreases), it is also clear that part of the between- and within-species variability has to be ascribed to variability in β^C^ and associated changes in *R*_bs_ and *R*_si_.

**Fig. 4:**
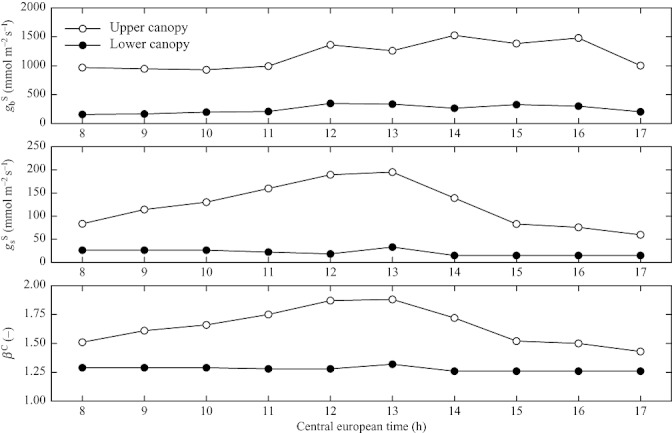
Diurnal time course of measured boundary layer (*g*_b_) and stomatal (*g*_s_) conductance to COS and the resulting β^C^ coefficient (assuming a constant *g*_i_^S^ of 0.3 mol m^−2^ s^−1^). Data are for *Trifolium pratense* measured on 4 June 2009 in a temperate mountain grassland in Austria. Upper and lower canopies refer to 0.35 and 0.05 m canopy height, respectively. For further details regarding the experiment, we refer to Wohlfahrt *et al*. (2010).

An example of how diurnal variability in *g*_b_^S^ and *g*_s_^S^ may affect β^C^, and thus in turn λ^C^, under field conditions, is given in Fig. 4 using boundary layer and stomatal conductance values measured over the course of 1 d in a temperate mountain grassland (Wohlfahrt *et al*. 2010). In this particular case, differences in β^C^ values between the upper and lower canopies (1.4–1.8 and 1.2–1.3, respectively) were driven mainly by differences in stomatal conductance (assuming a constant *g*_i_^S^ of 0.3 mol m^−2^ s^−1^), as *R*_bs_ values (6–21) were in a similar range in the upper and lower canopies. Assuming a χ_i_^C^/χ_a_^C^ ratio of 0.7 (Larcher 2001) results in λ^C^ values of 1.8–2.3 and 2.5–2.6 in the upper and lower canopies, respectively. On the other hand, assuming a constant *g*_i_^S^ is likely to be incorrect because experimental evidence suggests CA activity, which is implicit in *g*_i_^S^, to be dependent on leaf cellular pH (Sültemeyer 1998), light intensity (Moskvin *et al*. 2000), as well as to be under circadian control (Eriksson *et al*. 1998).

Another complicating issue, that up to now has not received appropriate attention, is whether λ^C^ determined from measurements in well-ventilated leaf enclosures is applicable to real-world, within-canopy transfer processes. Firstly, within plant canopies boundary layer conductances are not necessarily large as opposed to stomatal conductances (Baldocchi 1988), for example, combination of low wind speeds and vigorous transpiration. Secondly, transfer mechanisms across the boundary layer may be dominated by free instead of forced convection with light winds and strong leaf-to-air temperature gradients (Finnigan & Raupach 1987), which would change the exponent in Eqn 4 to 0.75 (Campbell & Norman 1998).

Both the variability in λ^C^ inferred from theoretical considerations (Fig. 2) and experimental evidence shown in Figs 3 and 4 suggest λ^C^ to differ between plant species and vary dynamically in response to changing environmental conditions. This conclusion seems to be inconsistent with constant λ^C^ values in the range of 2–3 reported in previous studies (Sandoval-Soto *et al*. 2005; Seibt *et al*. 2010). On the basis of Eqns 10 and 11, we suggest λ^C^ values on the order of 2–3 to reflect similar experimental conditions rather than an underlying universal principle. Most of the studies to date have been performed at leaf level under laboratory conditions by using enclosure systems [see review by Sandoval-Soto *et al*. (2005)] and thus tend to be biased towards high boundary layer (due to leaf enclosures usually being well ventilated) and relatively low stomatal conductances (due to limitations resulting from pot size and growth under relatively low light conditions). For example, for an infinite *R*_bs_ and a χ_i_^C^/χ_a_^C^ ratio of 0.7, λ^C^ values between 2 and 3 are observed for *R*_si_ < 0.4. Limited support for this hypothesis derives from the recent work of Sandoval-Soto *et al*. (2005), where field as opposed to laboratory studies tended to yield higher λ^C^ values. If true, the reliability of available λ^C^ values for estimating canopy net photosynthesis under field conditions may be seriously questioned.

### Transferring the leaf-level COS–CO_2_ relationship to the canopy level

The ecosystem level net exchange of COS (*F*_e_^S^) comprises, in addition to the leaf exchange, flux contributions by above- and below-ground sources/sinks, that is


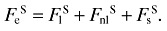
(12)

Here, *F*_nl_^S^ represents above-ground COS flux contributions other than from leaves (i.e. woody organs, flowers, attached dead plant matter, etc.), and *F*_s_^S^ represents COS fluxes from/to the soil surface. Both COS emission from and uptake by soils have been reported, although COS emissions from soils in some older studies appear to be due to experimental artefacts caused by the use of COS-free instead of ambient air in soil enclosures (Watts 2000). Recent studies, where soil chambers were flushed with ambient air, have reported soil COS uptake rates relative to deposition to vegetation that ranged from negligible (Xu *et al*. 2002; White *et al*. 2010) to dominant (Kuhn *et al*. 1999). Velmeke (1993), cited in Xu *et al*. (2002), investigated COS exchange in branches with and without leaves and found deposition and emission of COS, respectively. Given the scarcity of ecosystem-scale COS flux measurements (Xu *et al*. 2002), the significance of soil and non-leaf COS exchange is thus unclear and *F*_nl_^S^ + *F*_s_^S^ in Eqn 12 should not be neglected *a priori*. Ideally, *F*_nl_^S^ + *F*_s_^S^ would be quantified concurrently with *F*_e_^S^ in order to derive canopy-scale *F*_l_^S^ by difference. Such an approach may however be problematic in practice, as there is a mismatch in footprint of soil/branch enclosure as opposed to above-canopy micrometeorological flux measurements, which may be aggravated by the presence of spatial heterogeneity in COS sources/sinks. In addition, concurrent measurements of *F*_nl_^S^ + *F*_s_^S^ and *F*_e_^S^ would significantly increase the experimental effort. As an alternative, night-time measurements of *F*_e_^S^ might be used for estimating daytime *F*_nl_^S^ + *F*_s_^S^, similar to the current practice of estimating daytime ecosystem respiration from night-time CO_2_ flux measurements (Wohlfahrt *et al*. 2005). For this approach to work, night-time leaf COS exchange should be negligible, as confirmed by Sandoval-Soto *et al*. (2005), who found COS uptake during darkness to virtually cease when stomata are closed. In contrast, White *et al*. (2010) reported significant COS uptake by loblolly pine during darkness. Micrometeorological night-time COS flux measurements may however be problematic due to methodological limitations during calm and stable atmospheric conditions (Aubinet 2008). It remains to be determined whether approaches for dealing with unreliable night-time measurements developed for CO_2_, for example, filtering of data according to the magnitude of turbulence and imputation of resulting gaps based on empirical regression models (Goulden *et al*. 1996; Gu *et al*. 2005) are applicable to COS as well.

As mentioned earlier, only a handful of concurrent ecosystem-scale COS and CO_2_ flux measurements have been published so far (Hofmann, Hofmann & Kesselmeier 1992; Bartell *et al*. 1993; Xu *et al*. 2002). In order to explore the magnitude and variability of ecosystem-scale λ^C^ and differences to leaf-scale values, we re-analysed COS, CO_2_ and H_2_O flux data collected by Xu *et al*. (2002) over a Norway Spruce forest in Germany using the relaxed eddy accumulation (REA) method. Due to the lack of concurrent measurements of *F*_nl_^S^ + *F*_s_^S^ and reliable night-time REA estimates of *F*_e_^S^, we have assumed *F*_e_^S^ = *F*_l_^S^. This simplification is supported by soil COS exchange measured at the same site by Steinbacher, Bingemer & Schmidt (2004) showing an average deposition of −0.81 ± 0.03 pmol m^−2^ s^−1^ to the soil as opposed to an average ecosystem deposition of −93 ± 11.7 pmol m^−2^ s^−1^ (Xu *et al*. 2002). Ecosystem COS flux measurements of Xu *et al*. (2002), however, were on average positive during the morning and the evening when the NEE was positive (i.e. net loss of CO_2_ to the atmosphere), suggesting that other COS sources (e.g. woody plant material) may be playing an important role. Disentangling canopy net photosynthesis from NEE measured above the canopy would require estimates of soil and woody respiration, which were not available for this study. However, by following a common practice (Wohlfahrt *et al*. 2005), we used night-time NEE measurements under windy conditions for parameterizing RECO as a function of air temperature. RECO was then extrapolated to daytime temperature conditions to derive GPP as GPP = NEE − RECO. We have used GPP as a proxy for canopy net photosynthesis, recognizing that canopy net photosynthesis is larger (i.e. less negative) than GPP by the amount of CO_2_ respired from autotrophic tissues during daylight (Larcher 2001). λ^C^ was then calculated separately for NEE and GPP by dividing the measured ecosystem-scale COS fluxes normalized with ambient COS mole fractions with the measured NEE (inferred GPP) normalized with ambient CO_2_ concentrations in analogy to Eqn 5.

**Fig. 5:**
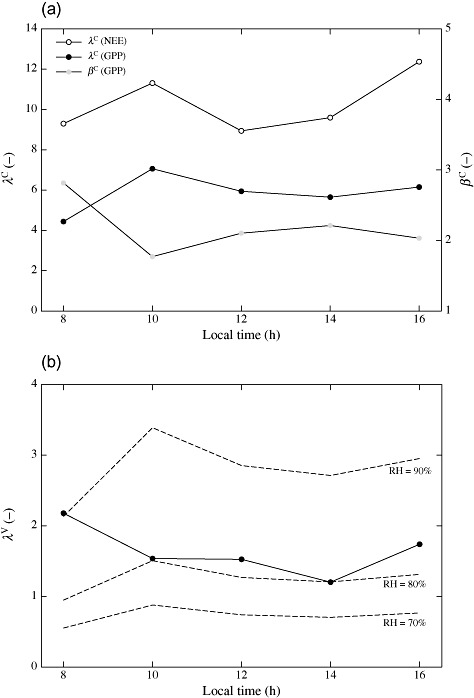
Ecosystem-scale λ^C^ (a) and λ^V^ (b) values re-calculated from bin-averaged CO_2_, H_2_O and COS flux measurements over Norway Spruce (Xu *et al*. (2002); note that periods with average net COS emission were excluded). GPP was calculated by parameterizing RECO, derived from night-time NEE measurements under windy conditions (horizontal wind speed > 3 m s^−1^), as a function of air temperature which was then extrapolated to daytime conditions as GPP = NEE − RECO. β^C^ values in (a) were calculated from measured λ^C^ values (based on GPP) and an χ_i_^C^/χ_a_^C^ ratio of 0.92 [Huber (1993) cited in Seibt *et al*. (2010)]. Dashed lines in (b) refer to λ^V^ at various RHs calculated from β^C^ shown in (a) and an average β^V^/β^C^ ratio of 1.5.

Values of λ^C^ calculated by considering NEE and GPP averaged 10.3 and 5.8 (Fig. 5), respectively. Considering that GPP overestimates canopy net photosynthesis, λ^C^ values resulted to be at least by a factor of 2 larger than the range of 2–3 reported by Sandoval-Soto *et al*. (2005) and Seibt *et al*. (2010) based on leaf-level laboratory enclosure studies. On a diurnal timescale, λ^C^ varied from 9 to 12.4 (38%) and from 4.4 to 7.1 (59%) for NEE and GPP, respectively. The range of λ^C^ variability is comparable in magnitude to the one deduced from diurnal changes in *g*_b_^S^ and *g*_s_^S^ (31%; Fig. 3), directly supporting our idea that changes in *g*_b_^S^, *g*_s_^S^ (and most likely *g*_i_^S^) cause diurnal variability in β^C^ and thus in λ^C^. With known λ^C^ values, Eqn 10 can be used to explore possible values for β^C^ and χ_i_^C^/χ_a_^C^. Commonly observed χ_i_^C^/χ_a_^C^ ratios between 0.5 and 0.8 resulted in β^C^ values of 0.35–0.87, well below the theoretical limit of 1.14 calculated from Eqn 9. In order to reach β^C^ values within the theoretical range (Fig. 2), χ_i_^C^/χ_a_^C^ values of at least 0.85 are required. While χ_i_^C^/χ_a_^C^ values > 0.8 are higher than the usual range (Larcher 2001), Huber (1993), cited in Seibt *et al*. (2010), indeed determined χ_i_^C^/χ_a_^C^ values of 0.91–0.93 for Norway Spruce, resulting in β^C^ of 1.9–2.5 (Fig. 5), that is, clearly within the range shown in Fig. 2.

## ESTIMATING CANOPY TRANSPIRATION FROM COS EXCHANGE MEASUREMENTS

Although several authors have linked ecosystem H_2_O and COS flux measurements (Hofmann *et al*. 1992; Xu *et al*. 2002), to our knowledge only one study (Seibt *et al*. 2010) explicitly mentioned the potential of COS as a tracer for estimating canopy transpiration. This may be due to the fact that the diffusion pathway of COS has less in common with H_2_O than CO_2_ (Fig. 1) and COS may thus be anticipated to be less suitable as a tracer for H_2_O.

In analogy to Eqn 10, the following link can be established between transpiration and the COS deposition velocity:


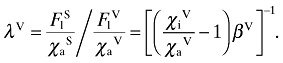
(13)

The coefficient β^V^ can be calculated in analogy to Eqn 9 but does not equal β^C^ because of differences in the COS to CO_2_ and H_2_O diffusivity ratios. The ratio of β^V^/β^C^ varies between *ca.* 1.4 and 1.7, depending on *R*_bs_.

Provided leaf temperature and atmospheric pressure (required to estimate the saturation water vapour mole fraction χ_i_^V^) are available and assuming a known value for β^V^ (our previous discussion on β^C^ applies here as well), Eqn 13 can in principle be used to infer transpiration on the basis of a measured COS deposition velocity. While leaf temperature is usually known with enough precision in leaf enclosures [but see Sharkey, Wiberley & Donohue (2008)], this is not granted at canopy scale under field conditions. Therefore, we further develop Eqn 13 according to Penman (1948) by introducing *T*_1_ = *T*_a_ + Δ*T*, where *T*_a_ represents air temperature and Δ*T* the difference between *T*_l_ and *T*_a_. As a consequence, χ_i_^V^/χ_a_^V^ in Eqn 13 can now be reformulated as:


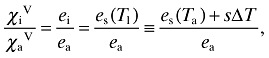
(14)

which holds for small values of Δ*T* and where *e* represents the vapour pressure and *s* the slope of the saturation vapour pressure function (Campbell & Norman 1998). Combining Eqns 13 and 14 yields, after some re-arrangement, the following expression:


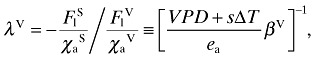
(15)

where VPD refers to the vapour pressure deficit of air, that is, *e*_s_*(T*_a_*)-e*_a_.

Equation 15 clearly addresses the relationship between transpiration and COS deposition velocity showing how that relationship may be sensibly affected by changes in Δ*T*, *T*_a_ and *e*_a_, independently of β^V^. If Δ*T* = 0 (isothermal case), the right-hand side of Eqn 15 reduces to ((RH^−1^ − 1) β^V^)^−1^, scaling positively with RH. When Δ*T* ≠ 0, additional non-linear variability proportional to Δ*T* arises because *s* increases exponentially with temperature (Campbell & Norman 1998).

In contrast to the χ_i_^C^/χ_a_^C^ ratio, which appears relatively conservative under a wide range of environmental conditions (Larcher 2001), the ratio of the saturation vapour pressure (at leaf temperature) to ambient vapour pressure may be expected to be much more variable, causing larger variability in λ^V^ as compared with λ^C^. Radiometric leaf temperature measurements would greatly reduce this problem in principle, may though be difficult in practice due to contributions by non-transpiring components such as the soil surface and/or woody plant tissue (Sánchez *et al*. 2009). In addition, radiometric measurements of leaf temperature may not necessarily represent a good estimate to be used for calculating the intercellular saturation vapour pressure due to within-canopy differences in the sources of thermal radiation and latent heat (Campbell & Norman 1998).

Because to our knowledge no data regarding λ^V^ have been published so far, we again make use of the study of Xu *et al*. (2002) to investigate the magnitude and diurnal variability of λ^V^ at ecosystem scale. As shown in Fig. 5, λ^V^ results to have an average value of 1.64 (ranging from 1.2 to 2.2), exhibiting higher and lower values in the morning/evening and noon, respectively, as expected from the positive relationship with RH and the typically lower RH values around noon. Assuming a constant β^V^/β^C^ ratio, the additional variability in λ^V^ (81%) as opposed to GPP-based λ^C^ (59%) is due to diurnal variability in the χ_i_^V^/χ_a_^V^ ratio, supporting our arguments raised previously.

## ESTIMATING CANOPY STOMATAL CONDUCTANCE FROM COS EXCHANGE MEASUREMENTS

By re-arranging Eqn 3, leaf stomatal conductance to COS, which may be converted to CO_2_ or H_2_O via Eqn 4, may be calculated as:


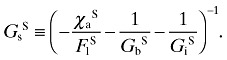
(16)

Equation (16) differs from the equations developed above for canopy net photosynthesis and transpiration in three different aspects: (1) the COS to CO_2_ (λ^C^) and H_2_O (λ^V^) deposition velocity ratios are not included; (2) instead, Eqn 16 contains two unknowns as absolute numbers – the *G*_b_^S^ and the *G*_i_^S^; (3) *G*_b_^S^ and *G*_i_^S^ represent bulk quantities: *G*_b_^S^ includes both the aerodynamic and the quasi-laminar boundary layer conductance (Monteith & Unsworth 1990), while *G*_i_^S^ is the integral of the internal conductance over the canopy leaf area – in order to emphasize this difference to the leaf-scale conductances used in Eqns 1–4, 7–11, we have used uppercase letters in Eqn 16. While models for the combined aerodynamic and quasi-laminar boundary layer conductance are available [but see Liu *et al*. (2007) for systematic uncertainties], little *a priori* knowledge is usually available on the magnitude of *g*_i_^S^, which is *inter alia* why λ^C^ and λ^V^ were introduced earlier, prohibiting a reliable up-scaling to *G*_i_^S^. A better quantitative understanding of *g*_i_^S^ (cf. Stimler *et al*. 2011) needs to be achieved to employ COS as a practical tool for estimating canopy stomatal conductance.

## CONCLUSIONS

The theoretical basis for the observed co-variation of leaf net photosynthesis, transpiration and COS uptake was reviewed. In addition, the assumptions which are required to use COS as a tracer for canopy net photosynthesis, transpiration and stomatal conductance, were discussed. Based on our analysis, we identified the following two priorities for future research:

1. We showed that λ^C^ and λ^V^ are not constants, but vary as a function of χ_i_^C^/χ_a_^C^ and χ_i_^V^/χ_a_^V^, respectively, and the ratios of boundary layer to stomatal and stomatal to internal conductance (Eqns 9–10 & 13). Due to the comparably more conservative nature of χ_i_^C^/χ_a_^C^ and the more similar diffusion pathway, our theoretical analysis suggests COS to represent a better tracer for CO_2_ than H_2_O. For routine application of Eqn 6 in field conditions, it will be necessary to develop a better understanding of the *in situ* variability and the factors controlling λ^C^ and λ^V^, which may result to be quite different from what has been observed under laboratory conditions by using leaf enclosures. To this end, it will be more important to study the four component processes controlling λ^C^ and λ^V^ in greater detail than limiting the investigation to the deposition velocities ratios. A particularly important step into this direction, which is prerequisite to improving the potential of COS as a tracer for canopy conductance, is to better understand and quantify variability in *g*_i_^S^ between species and on diurnal and seasonal time scales. A promising approach for independently characterizing λ^C^ under field conditions has already been put forward by Seibt *et al*. (2010), who showed that λ^C^ is related to the ^13^C discrimination during photosynthesis and that for example δ^13^C of leaf samples could be used as time-integrated estimates for λ^C^.
2. The potential of COS as a tracer for canopy-scale exchange processes hinges upon our ability to operationally disentangle leaf from any other ecosystem COS exchange. While progress has been made in understanding the factors which drive soil COS exchange (Kuhn *et al*. 1999; Van Diest & Kesselmeier 2008), we definitely need a better quantitative understanding of the contribution of soil and other non-leaf ecosystem components to the overall ecosystem-scale COS exchange. Here it appears that advantage can be taken from the vast amount of experience gathered in recent years in disentangling ecosystem CO_2_ flux components (Reichstein *et al*. 2005).

In summary, our study confirmed previous pioneering work highlighting the potential of COS as a tracer for canopy net photosynthesis; however, we have also indicated a number of limitations. For the first time, we have assessed the link between leaf COS and H_2_O exchange, addressing the potential and the limitations of COS as a tracer for canopy transpiration and stomatal conductance. At present, we face a serious lack of ecosystem-scale field measurements (Brühl *et al*. 2011) that represent an essential requirement for assessing the practical significance of these limitations and whether or not ecosystem COS flux measurements will be able to provide sensible constraints on canopy net CO_2_ and H_2_O exchanges. Such measurements will be of great value also for studies at larger scales (e.g. regional or global), which aim at inverting concurrent COS and CO_2_ concentration measurements to disentangle the components of the net ecosystem CO_2_ exchange (Campbell *et al*. 2008). We thus advocate concurrent measurements of ecosystem-scale COS, CO_2_ and H_2_O exchange and the corresponding component fluxes to allow testing and validation of our theoretical COS exchange models and their relation to canopy CO_2_ and H_2_O fluxes under field conditions. Here, great advancements can be expected from recent developments in analytical instrumentation (Graus *et al*. 2010; Stimler *et al*. 2010b) that will allow quantifying ecosystem-scale COS exchange by using the eddy covariance method in the near future.

## Abbreviations

*a*: coefficient in Eqn 4
CA: carbonic anhydrase
CO_2_: carbon dioxide
COS: carbonyl sulfide
*D*^X^: diffusivity of CO_2_ (*D*^C^), H_2_O (*D*^V^) or COS (*D*^S^) in air
*e*_X_: ambient (*e*_a_) or internal (*e*_i_) water vapour pressure (kPa)
*F*_e_^S^: ecosystem-scale flux of COS (pmol m^−2^ s^−1^)
*F*_l_^X^: leaf flux of CO_2_ (*F*_l_^C^, µmol m^−2^ s^−1^), H_2_O (*F*_l_^V^, mmol m^−2^ s^−1^) or COS (*F*_l_^S^, pmol m^−2^ s^−1^)
*F*_nl_^S^: non-leaf above-ground flux of COS (pmol m^−2^ s^−1^)
*F*_s_^S^: soil flux of COS (pmol m^−2^ s^−1^)
*G*_b_^S^: combined aerodynamic and quasi-laminar boundary layer conductance for COS (mol m^−2^ s^−1^)
*g*_b_^X^: leaf boundary layer conductance for CO_2_ (*g*_b_^C^, mol m^−2^ s^−1^), H_2_O (*g*_b_^V^, mol m^−2^ s^−1^) or COS (*g*_b_^S^, mol m^−2^ s^−1^)
*g*_ch_^C^: chloroplast conductance for CO_2_ (mol m^−2^ s^−1^)
*g*_gp_^X^: gas phase conductance for CO_2_ (*g*_gp_^C^, mol m^−2^ s^−1^) or COS (*g*_gp_^S^, mol m^−2^ s^−1^) in the intercellular space
*G*_i_^S^: canopy-scale internal conductance for COS (mol m^−2^ s^−1^)
*g*_i_^X^: leaf internal conductance to CO_2_ (*g*_i_^C^, mol m^−2^ s^−1^) or COS (*g*_i_^S^, mol m^−2^ s^−1^)
GPP: gross photosynthesis (µmol m^−2^ s^−1^)
*G*_s_^S^: canopy-scale stomatal conductance for COS (mol m^−2^ s^−1^)
*g*_s_^X^: leaf stomatal conductance to CO_2_ (*g*_s_^C^, mol m^−2^ s^−1^), H_2_O (*g*_s_^V^, mol m^−2^ s^−1^) or COS (*g*_s_^S^, mol m^−2^ s^−1^)
*g*_w_^X^: combined cell wall, plasma membrane and cytosol conductance for CO_2_ (*g*_w_^C^, mol m^−2^ s^−1^) or COS (*g*_w_^S^, mol m^−2^ s^−1^)
H_2_O: water vapour
H_2_S: hydrogen sulfide
NEE: net ecosystem CO_2_ exchange (µmol m^−2^ s^−1^)
*R*_bs_: ratio of leaf boundary layer to stomatal conductance for COS
RECO: ecosystem respiration (µmol m^−2^ s^−1^)
RH: relative humidity (fraction)
*R*_si_: ratio of leaf stomatal to internal conductance for COS
*s*: slope of water vapour saturation function (°C^−1^)
*T*_a_: air temperature (°C)
*T*_l_: leaf temperature (°C)
VPD: vapour pressure deficit (kPa)
β^X^: beta coefficient for CO_2_ (β^C^) or H_2_O (β^V^)
Δ*T*: difference between air and leaf temperature (°C)
λ^X^: ratio of deposition velocities of COS to CO_2_ (λ^C^) or COS to H_2_O (λ^V^)
χ_a_^X^: ambient CO_2_ (χ_a_^C^, µmol mol^−1^), H_2_O (χ_a_^V^, mmol mol^−1^) or COS (χ_a_^S^, pmol mol^−1^) mole fraction
χ_c_^C^: chloroplast CO_2_ (µmol mol^−1^) mole fraction
χ_cs_^X^: chloroplast surface CO_2_ (χ_cs_^C^, µmol mol^−1^) or COS (χ_cs_^S^, pmol mol^−1^) mole fraction
χ_i_^X^: CO_2_ (χ_i_^C^, µmol mol^−1^), H_2_O (χ_i_^V^, mmol mol^−1^) or COS (χ_i_^S^, pmol mol^−1^) mole fraction in the sub-stomatal cavity
χ_w_^X^: cell wall CO_2_ (χ_w_^C^, µmol mol^−1^) or COS (χ_w_^S^, pmol mol^−1^) mole fraction
